# Two-Parameter Elliptical Fitting Method for Short-Cavity Fiber Fabry–Perot Sensor Interrogation

**DOI:** 10.3390/s19010036

**Published:** 2018-12-22

**Authors:** Xiongxing Zhang, Wei Wang, Haibin Chen, Ying Tang, Zhibo Ma, Kening Wang

**Affiliations:** 1 School of Optoelectronic Engineering, Xi’an Technological University, Xi’an 710021, China; zhangxiongxing@xatu.edu.cn (X.Z.); chenhaibin@xatu.edu.cn (H.C.); tangying6421@163.com (Y.T.); wangkening@xatu.edu.cn (K.W.); 2 Key Lab of Micro/Nano Systems for Aerospace, Ministry of Education, Xi’an 710072, China; zbma@nwpu.edu.cn; 3 Shaanxi Key Lab of MEMS/NEMS, Northwestern Polytechnical University, Xi’an 710072, China

**Keywords:** elliptical fitting method, fiber Fabry–Perot sensor, white light interference, short cavity

## Abstract

To solve the cavity interrogation problem of short cavity fiber Fabry–Perot sensors in white light spectral interrogation with amplified spontaneous emissions (ASEs) as the white light sources, a data processing method, using an improved elliptical fitting equation with only two undetermined coefficients, is proposed. Based on the method, the cavity length of a fiber Fabry–Perot sensor without a complete reflection spectrum period in the frequency domain can be interrogated with relatively high resolution. Extrinsic fiber Fabry–Perot air-gap sensors with cavity lengths less than 30 μm are used to experimentally verify the method, and are successfully interrogated with an accuracy better than 0.55%.

## 1. Introduction

Fiber Fabry–Perot (FP) cavities can be used as sensors for the measurement of various physical parameters, such as temperature [1], pressure [2], strain [3], acoustic waves [4], magnetic field [5], etc. Due to their advantages of high accuracy, high resolution, simple structure, miniature size, and immunity to electromagnetic interference, fiber FP sensors have attracted more and more attention from international researchers and companies. Notably, by using heat-resistant materials, like sapphire or SiC, to fabricate extrinsic FP sensing elements, fiber FP sensors can be used for applications in harsh environments or applications where conventional electronic or fiber sensors cannot survive [6,7,8,9].

The most important technique for the real application of fiber FP sensors is cavity length interrogation. There are several kinds of methods for the cavity length interrogation of fiber FP sensors, such as single wavelength interrogation [10], dual wavelength interrogation [11], and white light interference interrogation [12,13,14,15,16,17,18,19,20,21,22]. For single wavelength interrogation, a continuous-wave laser with single wavelength output is used, which has to be stabilized at the Q-point of the fiber FP cavity, and only relative cavity length changes in a limited range can be measured. Furthermore, there exists a directional ambiguity problem for this kind of interrogation. For dual wavelength interrogation, two lasers with appropriately different wavelengths are used to generate quadrature (90° phase difference) signals for the fiber FP cavity, which can unambiguously determine the changing direction of the FP cavity length and extend the measuring range remarkably. However, like single wavelength interrogation, only relative changes can be measured; absolute cavity lengths of fiber FP sensors cannot be extracted.

Given that accurate absolute cavity length can be interrogated, white light interference interrogation has become the most important demodulation method for fiber FP sensor applications. In white light interference interrogation, a low coherence or broadband light source is used to illuminate the fiber FP sensor, and uses a reference FP cavity with a tunable cavity length to achieve a correlation signal between the two FP cavities, from which the cavity length of the fiber FP sensor can be obtained. This process is called white light scanning correlation interrogation [12]. Additionally, an optical air or birefringent wedge can be used to directly obtain the correlation signal without temporal scanning, a process which is called spatial scanning correlation interrogation [13,14,15,16]. By introducing the phase shift technique into white light interferometry, the absolute optical path difference (OPD) in a fiber optic interferometer can be measured. This was also proposed for the absolute cavity length interrogation of fiber FP sensors, but with a relatively low length accuracy [17,18]. Another important and direct white light interference interrogation method, called white light spectral interrogation [19,20,21,22], uses an optical spectrum analyzer to directly determine the reflection spectrum of the fiber FP sensor and resolve the cavity length from the reflection spectrum.

In white light spectral interrogation, a data processing algorithm of a Fourier transform [19,20] or a digital correlation method [21,22] can be used to extract the absolute cavity length. The most commonly used optical sources in white light interference are amplified spontaneous emissions (ASEs) near the communication wavelength of 1550 nm; the 3-dB spectral width of such kinds of optical sources is usually below 50 nm. When a fiber FP sensor has a short cavity length below 30 μm, in a range of 50 nm near 1550 nm, the reflection spectrum in the frequency domain cannot have a complete period, which causes the Fourier transform or digital correlation method to fail. Thus far, there is no effective cavity length interrogation method for short cavity fiber FP sensors.

To solve the interrogation problem of short cavity fiber FP sensors, we think an elliptical fitting method [23] can be used, since it can be used for the calculation of the period of a sinusoidal signal when the length of the signal is limited without a complete period. The main weakness of an elliptical fitting method is that there are six undetermined coefficients for the building of an elliptical function. Usually we need to solve a six-order, overdetermined linear equation to obtain the six coefficients. The real-time performance is poor and the interrogating speed is low; thus, the method cannot be used in situations requiring high-speed cavity length interrogation, such as dynamic pressure sensing, acoustic or ultrasonic detection, vibration monitoring, etc.

In this paper, we propose a data processing method for the white light spectral interrogation of short cavity fiber FP sensors using an improved elliptical fitting equation with only two undetermined coefficients. A theoretical model of the method for short cavity fiber FP sensors is described in detail and it is also verified through real experiments.

## 2. Principle of FP Cavity Length Measurement

**Figure 1:**
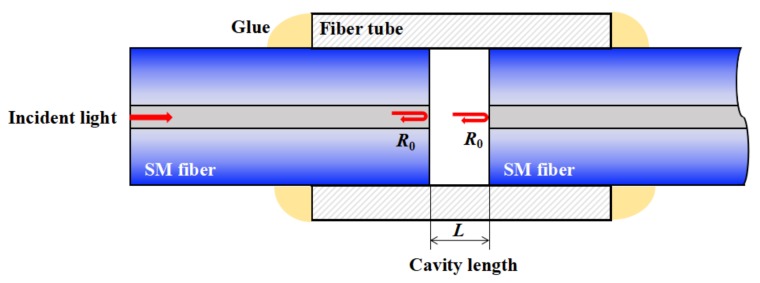
Typical structure of an extrinsic fiber Fabry–Perot (FP) sensor fabricated with two optical fibers and a short fiber tube. SM is single mode.

Fiber FP sensors can be fabricated using several kinds of methods, such as microelectromechanical systems (MEMS), femto-second (fs) laser writing, and the splicing and cutting method, but the basic structures of the fiber FP sensors are the same. A typical and simple extrinsic fiber FP interferometric (EFPI) sensor, fabricated with two optical fibers and a short fiber tube, is shown in Figure 1. This EFPI sensor is fabricated simply by inserting two optical fibers (single mode or multi-mode) into a fiber tube (or a glass capillary) with a short separation and then fixing them through gluing or laser welding. For any kind of fiber FP sensor, there are two parallel reflective surfaces to form an FP cavity.

If the reflectivity of one reflective surface is *R*_1_, the reflectivity of the other is *R*_2_, the cavity length is *L*, and the refractive index of the material filled in the cavity is *n*, then, for an optical wave with a wavelength of *λ*, the reflection ratio can be expressed by

(1) $$R\left(\lambda \right)=\frac{R_{1}+R_{2}+2\sqrt{R_{1}R_{2}}\cos\left(\frac{4\pi nL}{\lambda }\right)}{1+R_{1}R_{2}+2\sqrt{R_{1}R_{2}}\cos\left(\frac{4\pi nL}{\lambda }\right)}$$

in which loss caused by light diffraction during the back and forth propagation in the cavity of the EFPI sensor is neglected. It is well known that when a light emits from a limited aperture, it will be diffracted. In an external cavity formed by two fiber facets, the diffraction will prevent certain parts of the light from coming back to the fiber. However, for a short cavity length of only tens of micrometers, the diffraction loss is very low and can be ignored, which means Equation (1) can be safely used.

For a fiber FP cavity formed by two simple glass fibers, as shown in Figure 1, reflection ratios of the air–glass surfaces are relatively low, *R*_1_ = *R*_2_ = *R*_0_, *n* = 1, and the formula can be replaced by a two beam interference form in the wavelength domain

(2) $$R\left(\lambda \right)=2R_{0}\left(1+\cos\left(\frac{4\pi L}{\lambda }\right)\right)$$

or in the frequency domain

(3) $$R\left(f\right)=2R_{0}\left(1+\cos\left(\frac{4\pi L}{c}f\right)\right)$$

where *c* is the vacuum light speed. For a light with a wide spectral width from a low coherent source, such as a light emitting diode (LED), superluminescent LED (SLED), or an ASE incident on the fiber FP sensor, there will be several spectral peaks that appear in the reflection spectrum. Thus, we have

(4) $$2L=m\lambda_{m}=m\frac{c}{f_{m}}$$

in which m = 1, 2, 3…, *λ_m_* is the optical wavelength of each reflection peak, and *f_m_* is the optical frequency of each reflection peak.

Therefore, the relationship between the optical frequency and cavity length is

(5) $$f_{m}=m\frac{c}{2L}.$$

It can be found that in the frequency domain, the optical spectrum of an FP cavity is an equi-period signal, of which the period *T* (commonly called free spectral range) is

(6) $$T=f_{m+1}-f_{m}=\frac{c}{2L}.$$

Thus, the cavity length can be expressed as

(7) $$L=\frac{c}{2T}.$$

Then, Equation (3) can be rewritten as

(8) $$R\left(f\right)=2R_{0}\left(1+\cos\left(\frac{2\pi }{T}f\right)\right).$$

The main task of the interrogation algorithm of the Fourier transform, correlation, or our improved elliptical fitting method is to accurately determine the signal period from the reflection spectrum of the fiber FP cavity in the frequency domain, and then calculate the cavity length.

## 3. Six-Parameter Elliptical Fitting Method

In a two-dimensional Cartesian coordinate system, the general form of the elliptic equation is

(9) $$Ax^{2}+Bxy+Cy^{2}+Dx+Ey+F=0.$$

For a group of any *N* data points with coordinates of (*x_i_*, *y_i_*) on an elliptic curve, we can obtain the following linear equation of *A*, *B*, *C*, *D*, *E*, and *F*:

(10) $$Ax_{i}^{2}+Bx_{i}y_{i}+Cy_{i}^{2}+Dx_{i}+Ey_{i}+F^{\prime }=1$$

in which *i* = 1~N, *F*’ = *F* + 1; the six parameters *A*, *B*, *C*, *D*, *E*, and *F* can be solved using the matrix method. The above linear equations can be expressed in the matrix form as

(11) $$\left(\begin{array}{cccccc} x_{1}^{2} & x_{1}y_{1} & y_{1}^{2} & x_{1} & y_{1} & 1\\ x_{2}^{2} & x_{2}y_{2} & y_{2}^{2} & x_{2} & y_{2} & 1\\ \ldots & \ldots & \ldots & \ldots & \ldots & \ldots \\ x_{N}^{2} & x_{N}y_{N} & y_{N}^{2} & x_{N} & y_{N} & 1 \end{array}\right)\left(\begin{array}{c} A\\ B\\ C\\ D\\ E\\ F^{\prime } \end{array}\right)=\left(\begin{array}{c} 1\\ 1\\ \ldots \\ 1 \end{array}\right).$$

Let

(12) $$G_{N\times 6}=\left(\begin{array}{cccccc} x_{1}^{2} & x_{1}y_{1} & y_{1}^{2} & x_{1} & y_{1} & 1\\ x_{2}^{2} & x_{2}y_{2} & y_{2}^{2} & x_{2} & y_{2} & 1\\ \ldots & \ldots & \ldots & \ldots & \ldots & \ldots \\ x_{N}^{2} & x_{N}y_{N} & y_{N}^{2} & x_{N} & y_{N} & 1 \end{array}\right)$$

and

(13) $$H_{6\times 1}=\left(\begin{array}{c} A\\ B\\ C\\ D\\ E\\ F \end{array}\right)$$

where *G*_*N*×6_ is a column full rank matrix; then, Equation (11) can be rewritten as

(14) $$G_{N\times 6}H_{6\times 1}=E_{N\times 1}.$$

Obviously, Equation (14) is an overdetermined system of linear equations. By left multiplying both sides by (*G*_*N*×6_)*^T^*, we obtain

(15) $${\left(G_{N\times 6}\right)}^{T}G_{N\times 6}H_{6\times 1}={\left(G_{N\times 6}\right)}^{T}E_{N\times 1}.$$

Let *U*_6×6_ = (*G_N_*_×6_)*^T^* *G_N_*_×6_ and *W*_6×1_ = (*G_N_*_×6_)*^T^* *E_N_*_×1_, then it can be written as

(16) $$U_{6\times 6}H_{6\times 1}=W_{6\times 1}.$$

By solving the sixth-order square matrix *U*−1 6 × 6, the six parameters *A*, *B*, *C*, *D*, *E*, and *F* can be determined.

Translated in frequency coordinate $R\left(f\right)=2R_{0}\left(1+\cos\left(2\pi f/T\right)\right)$ with a frequency shift of *f*_0_, the reflected signal can be expressed as

(17) $$R(f+f_{0})=2R_{0}(1+\cos(\frac{2\pi }{\mathrm{FSR}}(f+f_{0})))$$

**Figure 2:**
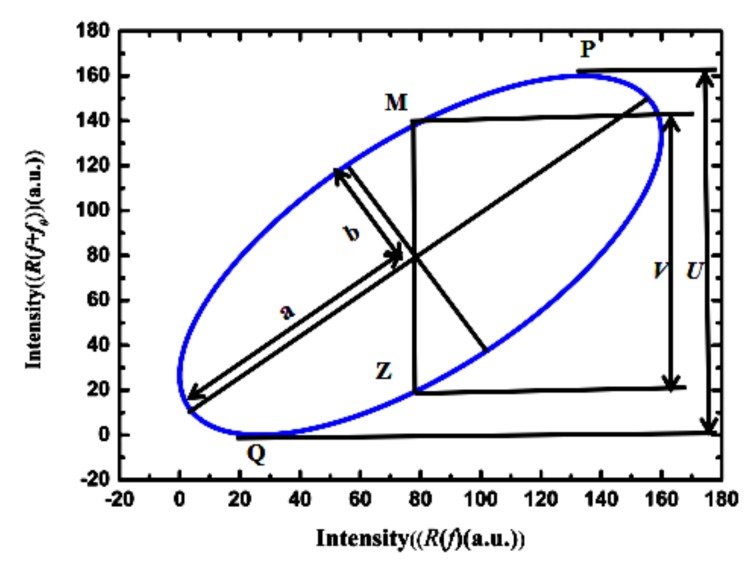
Lissajous figure obtained from the reflection spectrum and its translation in the frequency domain.

Using *R*(*f*) and *R*(*f + f*_0_) as the horizontal and vertical coordinates, respectively, a Lissajous figure can be obtained, as shown in Figure 2. The position of the ellipse center is determined by the direct current (DC) offset (2*R*_0_, 2*R*_0_). The positions of the two extreme points *P* and *Q* are (*x_p_*, *y_p_*) and (*x_Q_*, *y_Q_*), respectively.

Let the phase of *R*(*f*) be *π*/2. The two signals *R*(*f*) and *R*(*f + f*_0_) can be calculated by

(18) $$\left\{ \begin{array}{l} R(f)=2R_{0}\\ R(f+f_{0})=2R_{0}(1+\cos(\frac{2\pi }{T}f_{0})) \end{array}\right..$$

Equation (18) corresponds to two special points, *M* and *Z*, of the ellipse, as shown in Figure 2, of which the position coordinates are (*x_M_*, 2*R*_0_) and (*x_Z_*, 2*R*_0_), respectively. Suppose *V* is the distance between *M* and *Z*, and *U* is the distance between *P* and *Q*. Using the least-squares method to solve Equation (16) and obtain the six parameters *A*, *B*, *C*, *D*, *E*, and *F*, then *U* and *V* can be calculated by

(19) $$U=\frac{2\sqrt{4A^{2}C^{2}E^{2}-4ABC^{2}DE-A^{2}B^{2}C^{2}-AB^{2}CE^{2}+4AD^{2}C^{3}-16A^{2}C^{3}F+B^{3}CDE+8AB^{2}C^{2}F-B^{4}CF}}{C(4AC-B^{2})}$$

(20) $$V=\frac{4\sqrt{A^{2}E^{2}-ABED-4A^{2}CF+ACD^{2}+AB^{2}F}}{4AC-B^{2}}.$$

The phase difference *ϕ* between the two signals *R*(*f*) and *R*(*f + f*_0_) can be calculated by

(21) $$\phi =\tan^{-1}\left(\frac{U}{V}\right).$$

Then, the period *T* of the optical reflection spectrum of the FP sensor in the frequency domain is determined by

(22) $$T=\frac{2\pi }{\phi }f_{0}.$$

Considering Equation (7), the cavity length of the FP sensor can be expressed as

(23) $$L=\frac{c\phi }{4\pi f_{0}}.$$

## 4. Improved Elliptical Fitting Method for FP Interrogation

The problem with six-parameter elliptical fitting is that there exist six undetermined parameters to be determined, which makes the calculation very complex and time-consuming. If the six-parameter elliptical equation is changed into a two-parameter elliptical equation by coordinate translation and rotation, then data fitting on the basis of a two-parameter elliptical equation can be achieved, and the calculating complexity will be greatly reduced.

Since the signals *R*(*f*) and *R*(*f + f*_0_) have different phases but equal amplitude, the angle between the long axis *a* of the fitted ellipse and the X axis is always 45°. The center position of the ellipse is (2*R*_0_, 2*R*_0_). It is assumed that the original coordinate system is an XY coordinate system, and the transformed coordinate system is an X’Y’ coordinate system. Let the X’Y’ coordinate system rotate 45° with respect to the original coordinate system; then, the transformation formula of the XY coordinate system to X’Y’ is

(24) $$\left\{ \begin{array}{l} q=\frac{\sqrt{2}}{2}x+\frac{\sqrt{2}}{2}y\\ y^{\prime }=\frac{\sqrt{2}}{2}y-\frac{\sqrt{2}}{2}x \end{array}\right..$$

After the rotation, the center coordinate of the ellipse in the X’Y’ coordinate system is $\left(2\sqrt{2}R_{0},\;0\right)$. Then, through a horizontal translation of the ellipse to the origin of the coordinate by $x^{\prime }=q-2\sqrt{2}R_{0}$, the elliptic equation in the X’Y’ coordinate system will be in the form of

(25) $$\frac{x\prime^{2}}{A^{\prime^{2}}}+\frac{y\prime^{2}}{B^{\prime^{2}}}=1.$$

Suppose that the experimental data position in the new coordinate is (*x_j_*’, *y_j_*’); then, from Equation (25), we have

(26) $$\frac{{x_{j}^{\prime }}^{2}}{a^{\prime^{2}}}+\frac{{y_{j}^{\prime }}^{2}}{b^{\prime^{2}}}=1$$

in which *j* = 1~*n*. The matrix form can be expressed as

(27) $$\left(\begin{array}{cc} {x_{1}^{\prime }}^{2} & {y_{1}^{\prime }}^{2}\\ {x_{2}^{\prime }}^{2} & {y_{2}^{\prime }}^{2}\\ \ldots & \ldots \\ {x_{n-1}^{\prime }}^{2} & {y_{n-1}^{\prime }}^{2}\\ {x_{n}^{\prime }}^{2} & {y_{n}^{\prime }}^{2} \end{array}\right)\left(\begin{array}{c} \frac{1}{a^{\prime^{2}}}\\ \frac{1}{b^{\prime^{2}}} \end{array}\right)=\left(\begin{array}{c} 1\\ 1\\ \ldots \\ 1\\ 1 \end{array}\right).$$

Let $G_{n\times 2}^{\prime }=\left(\begin{array}{cc} {x_{1}^{\prime }}^{2} & {y_{1}^{\prime }}^{2}\\ {x_{2}^{\prime }}^{2} & {y_{2}^{\prime }}^{2}\\ \ldots & \ldots \\ {x_{n-1}^{\prime }}^{2} & {y_{n-1}^{\prime }}^{2}\\ {x_{n}^{\prime }}^{2} & {y_{n}^{\prime }}^{2} \end{array}\right)$ and $H_{2\times 1}=\left(\begin{array}{c} \frac{1}{a_{}^{\prime 2}}\\ \frac{1}{b^{\prime 2}} \end{array}\right)$; then. Equation (27) becomes

(28) $$G_{n\times 2}^{\prime }H_{2\times 1}^{\prime }=E_{n\times 1}$$

which is also an overdetermined system of linear equations. By left multiplying ${\left(G_{n\times 2}^{\prime }\right)}^{T}$, we have

(29) $${\left(G_{n\times 2}^{\prime }\right)}^{T}G_{n\times 2}^{\prime }H_{2\times 1}^{\prime }={\left(G_{n\times 2}^{\prime }\right)}^{T}E_{n\times 1}.$$

Let $U_{2\times 2}^{\prime }={\left(G_{n\times 2}^{\prime }\right)}^{T}G_{n\times 2}^{\prime }$ and $W_{2\times 1}^{\prime }={\left(G_{n\times 2}^{\prime }\right)}^{T}E_{n\times 1}$; then, Equation (29) can be rewritten as

(30) $$U_{2\times 2}^{\prime }H_{2\times 1}=W_{2\times 1}^{\prime }.$$

By solving the second-order square matrix ${\left(U_{2\times 2}^{\prime }\right)}^{-1}$, the two parameters *a*’ and *b*’ can be determined.

The phase difference *ϕ* between *R*(*f*) and *R*(*f + f*_0_) can be expressed as

(31) $$\varphi =2\tan^{-1}(\frac{b^{\prime }}{a^{\prime }}).$$

Then, through Equation (23), the cavity length of the FP sensor can be solved relatively easily with reduced calculating complexity and time spent.

## 5. Experiment and Discussion

**Figure 3:**
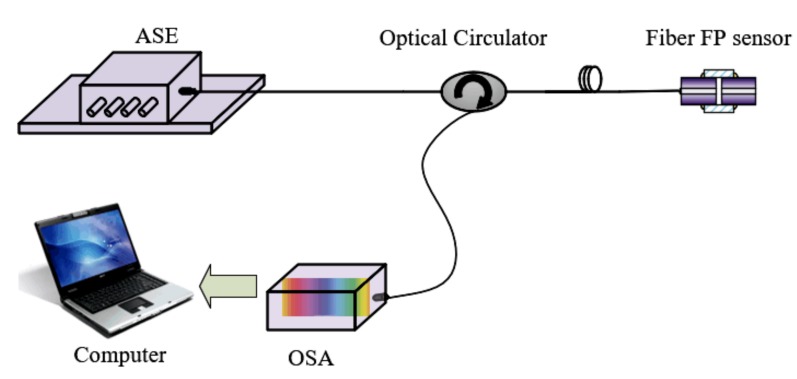
A white light fiber FP sensor spectral interrogation system. ASE is amplified spontaneous emission; OSA is optical spectral analyzer.

To verify the feasibility and performance of the improved elliptical fitting method for FP interrogation, a white light fiber FP sensor spectral interrogation system was built, which was composed of an ASE source, an optical circulator, a fiber FP sensor with an air gap, an optical spectral analyzer (OSA) module, an interrogation circuit, and a computer, as shown in Figure 3. The ASE source emitted light with a central wavelength of 1550 nm and a 3-dB bandwidth of 46 nm. The light was input into port 1 of the optical circulator and output from port 2 to the fiber FP sensor head with a short cavity, which reflected part of the light back into port 2 of the optical circulator, and exited from port 3 to the OSA module. The OSA module used was a high-speed spectrometer fabricated by Ibsen Photonics, which has a line InGaAs array of 256 pixels. The interrogation algorithm was written in a field-programmable gate array (FPGA) chip of a self-designed interrogation circuit, and the interrogated cavity length was sent through a universal asynchronous receiver/transmitter (UART) port to the upper computer to display in real time.

**Figure 4:**
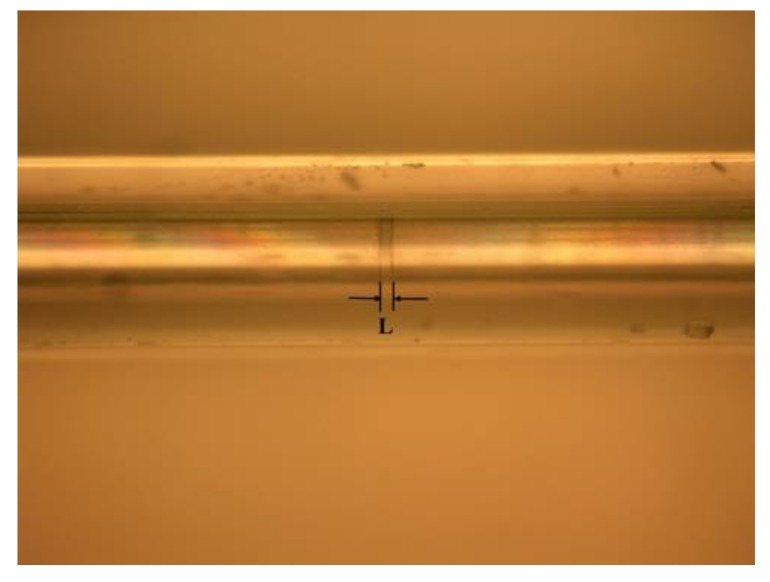
Photo of a fiber FP sensor.

The fiber FP sensor was fabricated by penetrating two standard single-mode fibers (SMF) into a glass capillary with an inner diameter of 128 μm. The two SMF fibers were cut and cleaved to form the short-cavity FP sensor. Firstly, one of the SMF fibers was penetrated into the glass capillary a short length, a little UV-cured adhesive was spread around the fiber cladding, then the SMF continued to penetrate with its facet near the middle position of the glass capillary and was illuminated with an ultraviolet lamp to fix the SMF and the glass capillary. Then the other SMF was penetrated into the glass capillary from the other end; the two parts were fixed on two high-precision three-axis translation stages and spread with a little UV-cured adhesive around the fiber cladding. The penetrating depth of the second SMF was tuned while being monitored by a wide band SLED (3-dB bandwidth of 90 nm) and a high-resolution OSA with a wavelength resolution of 30 pm. For the optical interference effect between the two facets of the two SMFs, there was a multi-peak form of a reflection spectrum on the OSA, and through the classical double peaks method, the separation between the two SMF facets was calculated. We finely tuned the translation stage and judged the distance through the reflection spectrum on the OSA. When a predetermined cavity length was achieved, the structure was illuminated again with an ultraviolet lamp to fix the second SMF in the glass capillary, and an air gap fiber FP cavity was formed. The separation between the two SMF facets was controlled to be shorter than 30 μm. We fabricated two short-cavity FP sensors with cavity lengths of 20.16 μm and 28.90 μm, respectively. Figure 4 shows a real photo of one fiber FP sensor.

The error of the cavity length ∆*L* determined through the double peaks method was calculated by

(32) $$\Delta L=\frac{c}{2T^{2}}\Delta T$$

in which ∆*T* is the uncertainty of the period of the reflection spectrum in the frequency domain, which was determined by the wavelength resolution of the OSA. For a wavelength near 1550 nm, ∆*T* = 3.75 GHz, for an air gap fiber FP cavity with a length of 20 μm, from Equation (32), the error or uncertainty of the calculated cavity length was less than 10 nm. The results were used as a reliable reference for examining the accuracy of our improved elliptical fitting method for FP interrogation.

**Figure 5:**
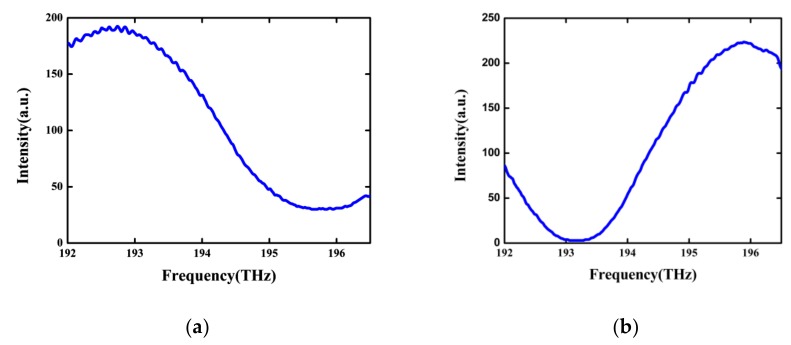
Reflection spectra of the fiber FP sensors for cavity lengths of (**a**) 20.16 μm and (**b**) 28.90 μm.

By demodulating through the interrogation system shown in Figure 3, it can be seen that for both of the FP sensors of short cavity length, the reflection spectra of the ASE light source in the frequency domain did not exceed one period, as seen in Figure 5.

**Figure 6:**
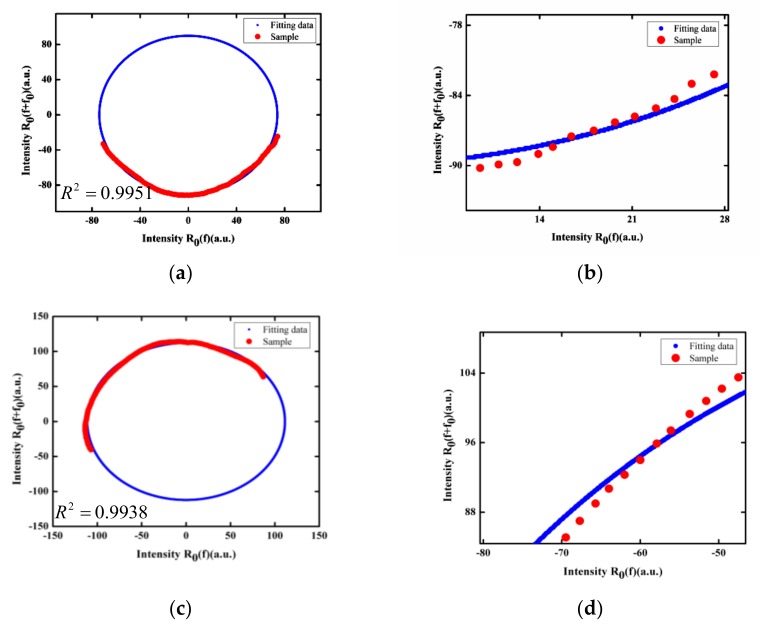
Improved elliptical fitting for the two fabricated fiber FP sensors. (**a**) Lissajous figure of the experimental data and fitting curve, (**b**) detailed view for the cavity length of 20.16 μm; (**c**) Lissajous figure of the experimental data and fitting curve, (**d**) detailed view for the cavity length of 28.90 μm.

Using the reflection spectral curve *R*_0_(*f*) in the frequency domain, and translating the spectrum along the frequency axis in the forward direction with a frequency displacement of *f*_0_, a new curve *R*_0_(*f + f*_0_) was obtained. When *f*_0_ = 1.596 THz, the relative intensity of the two curves at different frequencies was used as the horizontal and longitudinal coordinates, respectively, which were used as the data samples to be fitted, shown in Figure 6 as red points, which are only distributed on parts of the ellipse. Using our improved elliptical fitting method to fit the data, ellipse curves were produced for both of the two FP sensors, as shown in Figure 6. The coefficients of determination for the two fittings were *R*^2^ = 0.9951 and *R*^2^ = 0.9938, respectively. From Equation (31), it can be determined that the phase difference for the first fiber FP sensor was *ϕ* = 78°29’50’’, and the calculated cavity length *L* was 20.24 μm. For the second fiber FP sensor, the calculated cavity length was 29.01 μm.

There are some deviations between the experimental data and the fitting curves, as seen from Figure 6. The reasons may include the following: Firstly, elliptical fitting requires the signal to be in a cosine form, but the reflection spectrum of the EFPI sensor was not a rigorous but rather an approximate cosine. Secondly, the light source used in the experiment was an ASE with a spectral flatness of about 1 dB, which resulted in some signal distortions in the reflection spectrum. The deviations affected the coefficient of determination *R*^2^ for the elliptical fitting and resulted in some errors in the cavity length calculation.

Compared with the six-parameter elliptical fitting method, the improved two-parameter elliptical fitting method reduced the computation cost, with only two undetermined parameters to solve. At the same time, the long axis and the short axis of the ellipse was used to calculate the phase difference, and thus the calculation of the complex *U* and *V* was no longer needed. Therefore, the real time performance of the algorithm is much better than that of the six-parameter elliptical fitting method. On the platform of an FPGA, when the driving clock has a frequency of 200 MHz, using the improved elliptical fitting method for cavity length interrogation, the interrogation speed can reach 20 kHz, but when the six-parameter elliptical fitting method is used, only 4 kHz can be reached.

## 6. Conclusions

For a short length FP fiber sensor with an equivalent optical path below 30 μm, limited by the 3-dB spectral width of the light source, the reflection spectrum in the frequency domain usually cannot exceed a full period. As a result, the conventional Fourier transform, double peaks, or correlation methods cannot be used for the interrogation of the absolute cavity length. To solve this problem, a short length FP fiber sensor interrogation method is proposed, in which an improved elliptical fitting algorithm with only two parameters is used, which simplifies the conventional six-parameter elliptical fitting algorithm, reduces the calculating complexity, and improves the calculating resolution. Through the experiment, two short length air-gap fiber FP sensors with cavity lengths of 20.16 μm and 28.90 μm, respectively were interrogated through the proposed method and were successfully interrogated with an accuracy better than 0.55%.
